# So Many Are “Few,” but so Few Are Also “Few” – Reduced Semantic Flexibility in bvFTD Patients

**DOI:** 10.3389/fpsyg.2020.00582

**Published:** 2020-04-03

**Authors:** Stefan Heim, Corey T. McMillan, Christopher Olm, Murray Grossman

**Affiliations:** ^1^Institute of Neuroscience and Medicine (INM-1), Research Centre Jülich, Jülich, Germany; ^2^Department of Psychiatry, Psychotherapy, and Psychosomatics, Medical Faculty, RWTH Aachen University, Aachen, Germany; ^3^JARA-Translational Brain Medicine, JARA, Aachen, Germany; ^4^Department of Neurology, Frontotemporal Degeneration Center, Perelman School of Medicine, University of Pennsylvania, Philadelphia, PA, United States

**Keywords:** logic, quantifier, learning, neurodegeneration, adaptation, Broca, frontal cortex

## Abstract

The processing of quantifier words such as “many” or “few” is a complex operation supported by a plastic fronto-parietal network predominantly in the left hemisphere. The internal reference criterion defining a quantifier (e.g., ≥50% for “many”) can be modified in a learning paradigm. Most interestingly, changing the criterion for one quantifier also leads to a change in the criterion for the untrained quantifier, i.e., a semantic restructuring effect, which is supported by Broca’s region in the left inferior frontal cortex. Here, we applied this paradigm to patients with the behavioral variant of fronto-temporal dementia (bvFTD) because they suffer from loss of cognitive flexibility, reduced ability to process quantities and their values, impaired reinforcement learning, and language comprehension deficits. The question was whether the patients would be able to perform the task, show direct learning of the new quantifier meanings, and exhibit cognitive flexibility in terms of semantic restructuring. Eleven bvFTD patients took part in two behavioral experiments. In Experiment 1, in a first baseline block, each individual’s criterion for “many” and “few” was assessed. In block 2, subjects received feedback about their decisions. Contrary to their initial notion, a proportion of 40% yellow circles was reinforced as “many.” In block 3, the effect of this training on their judgments of “many” and “few” was re-assessed. The group of bvFTD patients showed a learning effect for the new criterion trained for the quantifier “many,” but failed to generalize this criterion shift to the other quantifier “few.” Experiment 2 was similar to Experiment 1, but the patients were trained in Block 2 to judge 60% of circles as “few,” with no training for “many.” Again, there was an average learning effect for the trained quantifier “few” over the entire group, but no generalization to “many.” Since the patients were still able to perform the task and showed learning of “many” to direct feedback, the data suggest that the generalization process, rather than initial learning, is more vulnerable to fronto-temporal degeneration.

## Introduction

In natural language, quantifiers are verbal expressions denoting quantities (e.g., *seven, a dozen*), sets (e.g., *many, a few, all*), or relations of sets (e.g., *more than one third, less than half, the bigger part*). Some quantifiers refer to an explicitly stated criterion (e.g., 5 in *at least five*, or 50% in *half*) that makes a quantifier-containing statement easily verifiable (“I brought my five books”). Other quantifiers, in contrast, refer to an implicit criterion (or degree) which may vary in different contexts (e.g., Schöller and Franke, 2016; Schöller, 2017): *Many elephants* might be six, whereas *many microbes* are probably several thousands. Moreover, depending on individual experience and standards, these not explicitly defined criteria can vary substantially between persons: *Many jelly beans* could be 10 for one child but 100 for another child. There is substantial inter-subject variability for various quantifiers (e.g., Oaksford et al., 2002; Pezzelle et al., 2018), which is larger for quantifiers referring to a variable as compared to a fixed degree criterion (Shikhare et al., 2015).

Most interestingly, quantifier semantics is not necessarily fixed even when only one individual person’s judgment is considered in only one clearly defined situation. Based on the finding that linguistic processing preferences can be modified via explicit feedback in a decision task (McMillan et al., 2012), and considering the adaptation level theory by Helson (1948) which states that habituation to some intensity or magnitude can shift the internal reference frame, we developed a paradigm in which quantifier semantics could be modified (Heim et al., 2015, 2016). In this paradigm, participants were rewarded for accepting definitions of *many* or *few* (e.g., calling 40% of items already *many* items) that went outside the range of their spontaneous judgments. Importantly, there were generalization effects to the semantics of other quantifiers: Such a shift in the internal criterion for “many-ness” also had an effect on the individual’s notion of “few-ness” and vice versa even though such a generalization had not been requested, suggested or rewarded in the paradigm. The findings indicate that quantifiers for which the semantics is not fixed to an explicit number can gradually shift their meaning.

Quantifier processing takes place at the interface of formal logic and reasoning, lexical semantics, and numerical cognition (e.g., Szymanik, 2007; Troiani et al., 2009; Deschamps et al., 2015; for a discussion of experimental options to assess quantifier processing properly cf. Szymanik and Zajenkowski, 2009). It relies thus on a larger fronto-parietal brain network (e.g., McMillan et al., 2005, 2013; Heim et al., 2012, 2016; Wei et al., 2014) in which the parietal aspects are related to number/numerosity processing whereas the frontal regions support working memory, semantic evaluation of numerosities and the translation from non-verbal to verbal formats (cf. Dehaene et al. (2003) triple code model). When quantifier meaning is changed, i.e., when cognitive flexibility is required, it is the inferior frontal cortex that supports this re-interpretation and re-structuring (Heim et al., 2016).

One condition in which cognitive flexibility, semantic evaluation and reinforcement learning are all compromised is the behavioral variant of fronto-temporal dementia (bvFTD). Clinically, patients suffering from bvFTD exhibit socially inadequate behavior, reduced emotional flexibility (apathy, decline of sympathy and empathy), and difficulties with their inhibitory control (perseverative, stereotyped, or compulsive behaviors; for a review cf. Butler and Chiong, 2019). The latter may be associated with the careless over-spending of the patients’ money and with alternations of the patients’ eating behaviors. By the patients’ care-givers, these changes are seen as a lack of social warmth and increase of selfish behavior, reduced emotional responsiveness to cues from significant others, and inadequate social decorum (misjudged social distance, sexual and financial allusions to strangers, etc.), and are judged as a burden (Brioschi Guevara et al., 2015).

These clinical characteristics emerge from the following progressive alterations in the cognitive domain (for a recent, comprehensive review cf. Johnen and Bertoux, 2019). Executive functions (selective attention, strategic planning, and working memory decline (e.g., Rahman et al., 1999; Libon et al., 2007). Likewise, cognitive flexibility, adaptation to a situational context and re-evaluation of circumstances are becoming more and more difficult (e.g., Zakzanis, 1998; Ibañez and Manes, 2012; Carr et al., 2015). Also, the processing of rewards and values *per se* (Clark et al., 2018; Wong et al., 2018) is impaired. Whereas bvFTD patients seem to have maintained their memory abilities to recall the value of words used as rewards in a learning paradigm, they failed to apply that knowledge in overt behavior. Consequently, due to suboptimal use of reward information, reinforcement learning can become increasingly difficult (Strenziok et al., 2011). Likewise and the patients’ meta-cognitive abilities decline (Eslinger et al., 2005).

Together, cognitive difficulties can cause problems for the patients’ language processing abilities, in particular sentence comprehension (Ash et al., 2006; Murray et al., 2007; Peelle et al., 2007, 2008). Interestingly, the extent of the progressive degeneration of the frontal lobes in bvFTD is directly linked to the patients’ abilities to process sentences containing quantifier statements (McMillan et al., 2013).

This pattern of findings can be summarized as follows: (1) Patients suffering from bvFTD have difficulties in assessing values, amounts, and quantities, both in terms of positive and negative sanctions *from* their relevant social environment and in terms of the degree of their own behavior toward their social environment. (2) Alterations in emotional processing make the value of reward even less effective. (3) The patients’ language comprehension abilities are affected. (4) The former three aspects together cumulate (a) in a problem to profit in learning paradigms and (b) in a problem to process language containing words denoting quantities. (5) Finally, parts of their difficulties are directly related to gray matter loss in Broca’s region relevant for language comprehension and semantic flexibility (even though the overall degeneration is more wide-spread, also extending into the temporal lobes).

Consequently, the aim of this study was two-fold. First, we tested whether patients with bvFTD are still able to change their degree for “many” or “few” in an explicit learning context for that quantifier, using explicit feedback for reinforcement learning. Second, in case that explicit learning was still possible, we wanted to see whether a transfer of that direct learning effect to the untrained quantifier in the sense of generalization/implicit learning was possible. To this end, we recruited bvFTD patients and asked them to perform the quantifier truth value judgment task involving reinforcement learning studied earlier (Heim et al., 2015).

## Experiment 1: Shifting the Criterion for “Many”

### Methods

#### Participants

Eleven patients diagnosed with bvFTD at the *Penn FTD-Center* participated in both experiments. Their average age at first participation was 62.7 years (range 53–82). They had received 16.8 years of education (range 12–22 years) and presented with a Mini Mental State Examination (MMSE) score of 24.2 which indicates a level of functioning at the lowest border of the normal range (Creavin et al., 2016). All patients participated in an informed consent procedure that was approved by an Institutional Review Board convened at the University of Pennsylvania. The demographic data and performance in neuropsychological tests for memory and executive functions are reported in Table 1. Note that, since no generalization of the results in the present experiments to real-life scenarios were intended at this stage of the research program, metacognitive abilities (reported to be impaired in FTD patients in general: Eslinger et al., 2005) were not assessed.

**TABLE 1:** Demographic data of the bvFTD patients.

Patient	MMSE	Age	Sex	Years of education
1	26	56	M	16
2	27	82	M	18
3	22	66	M	12
4	24	68	M	16
5	23	61	F	16
6	30	68	M	22
7	29	53	M	18
8	24	58	M	20
9	23	64	M	14
10	16	63	M	18
11	25	55	M	18

*^1^The significance was tested one-tailed because of the hypothesis that a patient performing well in Experiment 1 would also do so in Experiment ^2^The correlation for reinforcement learning would have been significant also in a two-tailed test. Importantly, the correlation for the transfer effect even failed to reach significance in a one-tailed test, thus underscoring that there really was no systematic transfer in the two experiments for bvFTD patients.*

#### Procedure

**FIGURE 1:**
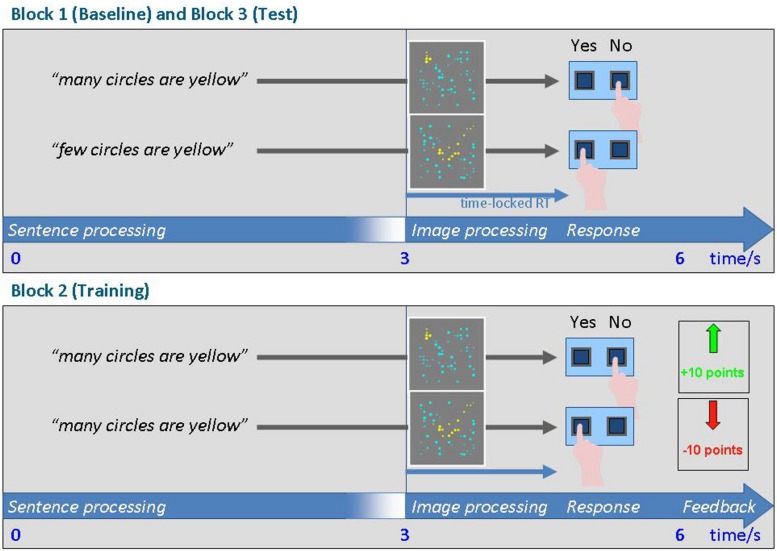
Schematic of the trials in blocks 1 and 3 **(top)** and in the adaptation block 2 **(bottom)**. Source: Heim et al. (2015). If so many are “few,” how few are “many”? Frontiers in Psychology, 6, 441. Copyright lies with the authors.

The procedures (cf. Figure 1) were identical to those previously used and reported by Heim et al. (2015) in the first proof-of-principle study and also in the subsequent fMRI study (Heim et al., 2016). Patients saw displays of blue and yellow circles on a gray background (Heim et al., 2012). Among the fixed number of 50 circles per display, proportions of yellow circles varied in steps from 20, 30, 40, 50, 60, and 70%. Each display was preceded by the sentence “*Many* of the circles are yellow” or “*Few* of the circles are yellow.” The patients were asked to judge whether the sentence adequately described the display by pressing one of two response buttons. The assignment of the YES and NO responses to the two response buttons was fixed for each single patient for the course of the entire experiment. Across patients, the assignment of YES or NO to the left or right button was varied.

The experiment consisted of three blocks. In the first block, we assessed the patients’ initial criteria for judging a given amount of circles as “*many*” or “*few*” (Block 1, of 378 trials counterbalanced for “few” and “many” trials along with the proportion of yellow circles). Next, reinforcement learning was applied in order to lower the criterion for “*many*” (Block 2, 162 trials). The patients gained points (positive feedback) if they responded YES to displays containing proportions of yellow circles of 40% or higher, or NO to displays with less 20 and 30% of yellow circles. They lost points (negative feedback) if they gave other responses. Finally, we tested whether this shift also had a repercussion for the criterion for “*few*” (Block 3, identical to Block 1) despite the fact that “few” had not featured in Block 2.

The timing of one trial in Block 1 and/or Block 3 is shown in Figure 1 (top). At the beginning of the trial, a written quantifier statement (“Many of the circles are yellow”/”Few of the circles are yellow”) was presented in the upper part of the screen for 3,000 ms. Next, the sentence remained on the screen on for 1,500 ms together with the stimulus picture containing yellow and blue circles, presented at the center of the screen for the same amount of time. The response options “YES” and “NO” were displayed in the lower part of the screen in order to remind the participant which button to press. Each trial ended with a blank screen for 1,500 ms. The participants’ responses were registered from the onset of the picture until the end of the trial.

The trial schema in Block 2 (i.e., the learning block) is shown in Figure 1 (bottom). Trials were similar to those in the baseline/test phase of Blocks 1/3, but additionally, feedback was given at the end of each trial. If the patient made a correct judgment (i.e., deciding that 40% of circles qualified as “many”), they gained 10 points. False responses were penalized by a loss of 10 points from their score. The feedback screen for correct responses showed a green arrow pointing upward and the information “+10” in green font. Feedback for errors was given as a downward red arrow and the text “-10” in red font. Patients started with an initial score of “+100.” The actual score was always displayed in the center of the upper portion of the screen.

All patients were familiarized with the stimuli and task in two short blocks prior to the actual experiment. The exact timing parameters can be found in Figure 1 (for details cf. Heim et al., 2015).

### Data Analysis

The patients’ acceptability judgments (i.e., their YES/NO responses) were aggregated per subject, experimental block, quantifier, and proportion of circles. Next, they were submitted to a 2 × 2 × 6 ANOVA with factors BLOCK (baseline/test), QUANTIFIER (many/few), and PROPORTION (20/30/40/50/60/70). Moreover, planned contrasts (Fisher test, one-tailed, Bonferroni-corrected) were calculated for the numbers of “yes” and “no” responses in blocks 1 and 3 at the critical proportion “40%” across participants, both for the trained and the untrained quantifier. The Fisher test was one-tailed because in the previous studies with this paradigm (Heim et al., 2015, 2016) and also with a modification of the paradigm (Heim et al., 2020) the direction of the effect was consistent in the same direction, i.e., toward the trained criterion.

### Results

The 2 × 2 × 6 ANOVA only yielded a significant interaction effect of QUANTIFIER × PROPORTION [*F*(5,6) = 4.521; *p* = 0.047]. All other effects failed to reach significance [main effect QUANTIFIER: *F*(1,10) = 0.497; *p* = 0.497; main effect BLOCK: *F*(1,10) = 0.846; *p* = 0.379; main effect PROPORTION: *F*(5,6) = 1.054; *p* = 0.466; interaction QUANTIFIER × BLOCK: *F*(1,10) = 0.061; *p* = 0.810; interaction PROPORTION × BLOCK: *F*(5,6) = 2.192; *p* = 0.184; interaction QUANTIFIER × PROPORTION × BLOCK: *F*(5,6) = 2.779; *p* = 0.123].

**FIGURE 2:**
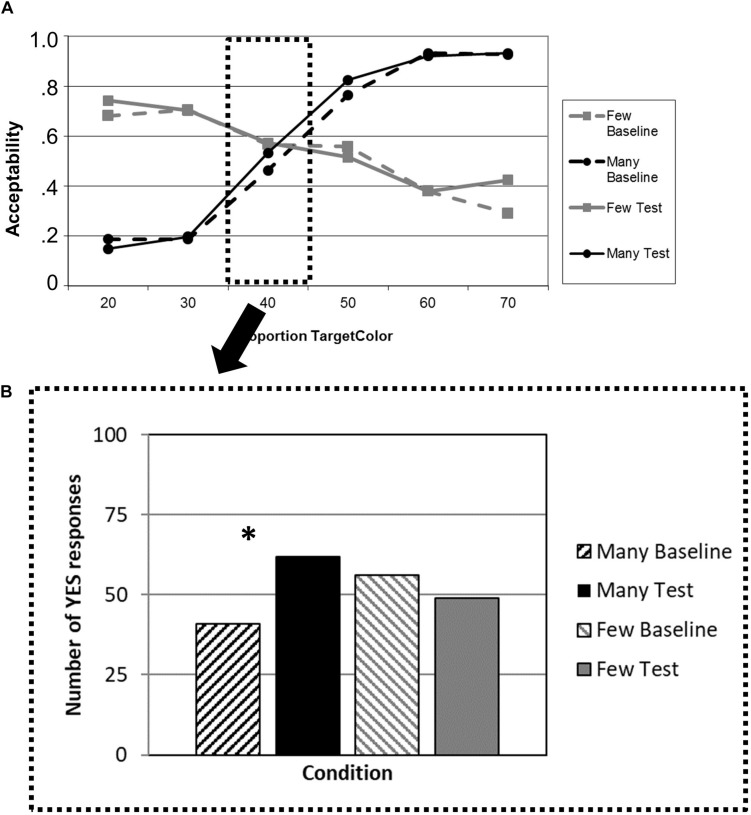
**(A)** Patients’ average acceptability ratings for a given proportion of circles of the mentioned color, plotted separately for the quantifiers “*many*” (black lines) and “*few*” (gray lines) in the baseline blocks (dashed lines) and the test blocks after adaptation (solid lines). **(B)** Number of “yes” responses for the critical proportion (40%) of circles of the mentioned color, plotted separately for “*many*” (black bars) and “*few*” (gray bars) in the baseline blocks (dashed bars) and the test blocks after adaptation (solid bars). ^∗^*p* < 0.05.

Testing the adaptation effect for “*many*” and “*few*” at the critical proportion “40%” illustrates this overall effect (Figure 2). We found a significant increase in the number of “yes” responses for “*many*” (Fisher test, one-tailed, *p* < 0.001), but no effect for “*few*” (*p* = 1). All *p*-values are Bonferroni-corrected for the number of comparisons (i.e., four).

### Discussion

Experiment 1 used the same setup for bvFTD patients as Heim et al. (2015) for healthy controls. The results differ both qualitatively and quantitatively from that earlier study. While the patient group showed on average an increase in the probability of accepting 40% of circles as “many” after the learning block, there was no indication of a generalization of that effect to the untrained quantifier “few.” In other words, while reinforcement learning was still possible (for discussion see Strenziok et al., 2011), a more general flexibility in the semantic system driven by that shift could not be observed. Even though no causal relationship can be established between the performance and atrophy of area 45 in the IFG, the pattern of result does not contradict the earlier neuroimaging findings by McMillan et al. (2013) and Heim et al. (2016). However, alternatively, it could just be a unique effect of the one quantifier “many” tested here. In order to exclude that possibility, Experiment 2 was conducted in which “few” instead of its polar opposite “many” was in the focus of learning.

## Experiment 2: Shifting the Criterion for “Few”

Experiments 1 and 2 were not run in one session, but the patients were re-invited to participate again after 4–22 weeks. Moreover, the patients completed the two experiments in a pseudo-randomized order, i.e., some first participated in Experiment 2 and the others in Experiment 1.

### Methods

The same patients as in Experiment 1 also completed Experiment 2, which was identical to Experiment 1 except for one variation: In block 2 of Experiment 2, the patients learned to judge proportions of 50 and 60% of circles as “*few*.” YES responses were reinforced for 60% or less yellow circles (patients gained 10 points), whereas other responses were sanctioned (patients lost 10 points). The proportions ranged between 30 and 80%. The analyses were analogous to those in Experiment 1.

### Results

The 2 × 2 × 6 ANOVA only yielded a significant interaction effect of QUANTIFIER × PROPORTION [*F*(5,6) = 4.818, *p* = 0.041] as well as significant main effects of QUANTIFIER [*F*(1,10) = 7.355, *p* = 0.022] and BLOCK [*F*(1,10) = 11.323, *p* = 0.007]. All other effects were non-significant [main effect PROPORTION: *F*(5,6) = 1.544, *p* = 0.304; interaction QUANTIFIER × BLOCK: *F*(1,10) = 0.255, *p* = 0.625; interaction PROPORTION × BLOCK: *F*(5,6) = 1.336, *p* = 0.363; interaction QUANTIFIER × PROPORTION × BLOCK: *F*(5,6) = 1.577, *p* = 0.296].

**FIGURE 3:**
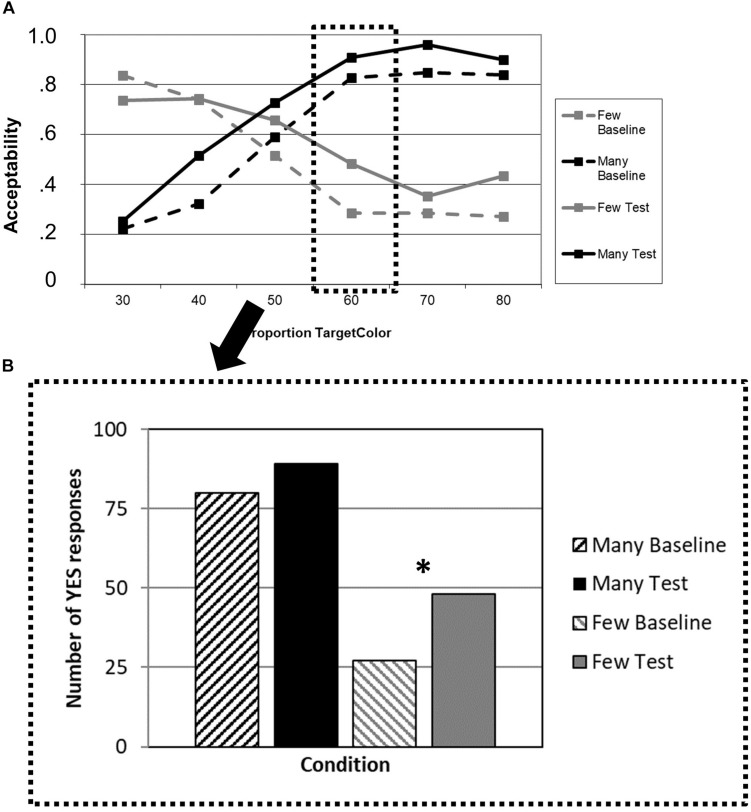
**(A)** Average acceptability ratings for a given proportion of circles of the mentioned color, plotted separately for the quantifiers “*many*” (black lines) and “*few*” (gray lines) in the baseline blocks (dashed lines) and the test blocks after adaptation (solid lines). **(B)** Number of “yes” responses for the critical proportion (60%) of circles of the mentioned color, plotted separately for “*many*” (black bars) and “*few*” (gray bars) in the baseline blocks (dashed bars) and the test blocks after adaptation (solid bars). ^∗^*p* < 0.05.

Testing the adaptation effect for “*many*” and “*few*” at the critical proportion “60%” illustrates this overall effect (Figure 3). We found a significant increase in the number of “yes” responses for “*few*” (*p* = 0.009, one-tailed) but no effect for “*many*” (*p* = 0.264, one-tailed). All *p*-values are Bonferroni-corrected for the number of comparisons (i.e., four).

### Discussion

The results of Experiment 2 are the expected mirror image of those obtained in Experiment 1. Again, the trained quantifier (in this case “few”) showed a direct effect of reinforcement learning into the intended direction, with higher proportions of circles being more likely accepted as “few.” Also, as in Experiment 1, the untrained quantifier “many” failed to show the generalization effect found in healthy controls. If anything, there was a numerical trend in the opposite direction, which, however, failed to reach significance. This duplicity of systematic patterns of results might be taken to suggest that there is really a connection between the bvFTD patients’ general brain atrophy (which, however, may also include their temporal lobes, thus not permitting lesion-symptom mapping-like conclusions), their performance in the truth value judgment task, and the underlying cognitive-semantic shifts (or their absence). The implications of the findings of Experiments 1 and 2 will be reflected together in the section “General Discussion.”

## Joint Analyses for Experiments 1 and 2

**FIGURE 4:**
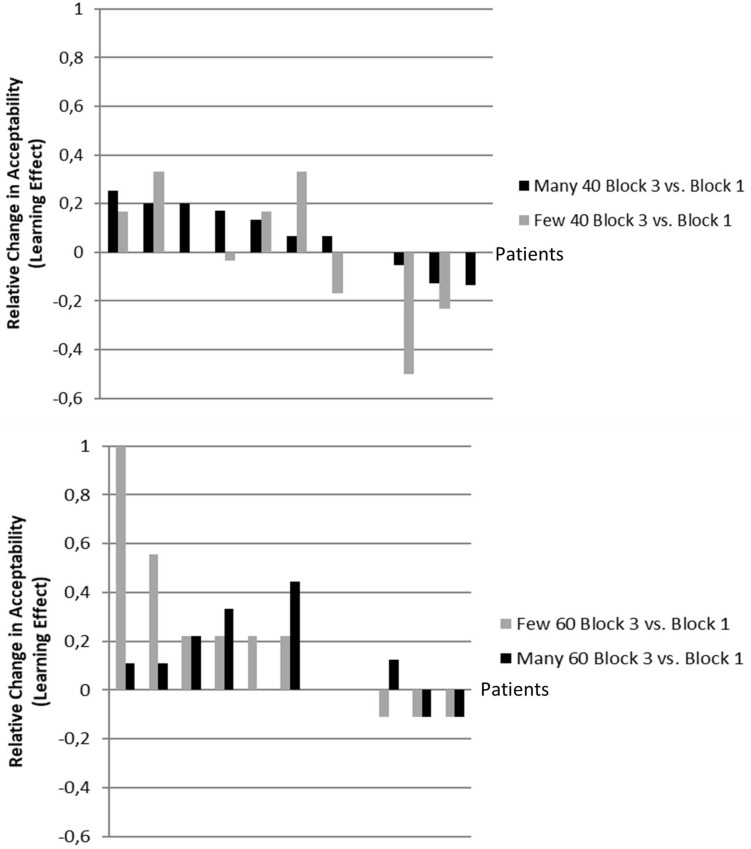
Individual learning for the trained (direct) and untrained (transfer) quantifier in Experiment 1 **(above)** and Experiment 2 **(below)**, sorted by the size of the direct reinforcement learning effect (above: black bars; below: gray bars). Each pair of bars represents one patient. If no change was found for a patient, i.e., in case the difference is zero, this patient’s bar/s is/are not appearing in the graph accordingly but leaves a gap.

Before discussing the results of Experiments 1 and 2, some further analysis shall be reported which establish a link between the direct and transfer learning effects in the two experiments. First, the individual learning performance was analyzed (Figure 4). It is evident that in both experiments not all of the patients (Experiment 1: *n* = 7 out of 11; Experiment 2: *n* = 6 out of 11) contributed to the group effect for direct reinforcement learning (Figure 4). At the same time, it is also evident that the size of the transfer learning effect for the respective untrained quantifier varies unsystematically with only a few patients exhibiting an effect in the expected direction (Experiment 1: *n* = 4; Experiments 2: *n* = 2), i.e., even though reinforcement learning took place, there were paradoxical effects for the generalization effect, which resulted in the reported non-significant generalization effect for the entire group.

**FIGURE 5:**
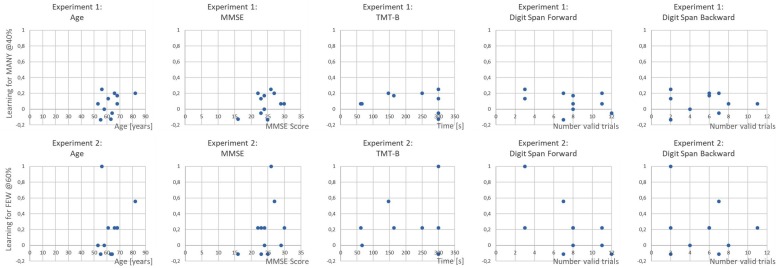
Scatterplots illustrating the relationship of the direct learning effects in Experiment 1 at proportion 40% **(upper panel)** and in Experiment 2 at proportion 60% **(lower panel)** with the patients demographic and neuropsychological data.

Next, in order to assess whether the learning effects in Experiments 1 and 2 were related, we ran additional correlation analyses for the trained and untrained quantifiers. Evidently, the size of the direct reinforcement learning in both experiments was very similar in size, resulting in a high correlation of *r* = 0.827 (*p* = 0.001 one-tailed). In contrast, the transfer effects, which were identified above as unsystematic, failed to show a significant relationship (*r* = 0.344; *p* = 0.150 one-tailed^1^). Finally, in Figure 5, the relationships of the direct learning effects and neuropsychological variables are displayed in order to complement the results and to provide a ground for subsequent studies.

## General Discussion

In two experiments we tested the hypothesis that bvFTD patients have reduced abilities to shift their sets of internal criteria of “many-ness” and “few-ness” over and above direct reinforcement learning. The overall pattern was exactly as expected. While the patient group on average still showed the direct learning effect, no transfer within the semantic system could be observed. These effects were consistent over the two experiments and across patients, with highly similar direct learning effects as indicated by the correlation analysis, and very unsystematic generalization. Finally, the MMSE score seemed to offer no explanation for the size of the learning effect.

This pattern of results is consistent with the previous fMRI study in healthy participants showing the involvement of the left IFG in the shifting of quantifier semantics (Heim et al., 2016) and quantifier processing in general (McMillan et al., 2005, 2013; Heim et al., 2012). Specifically for bvFTD, it also confirms earlier reports of semantic processing deficits in bvFTD patients (Cousins et al., 2016, 2017; for a review cf. Cousins and Grossman, 2017). Interestingly, these semantic processing deficits in language production and comprehension were observed outside the area of formal semantics (quantifier processing) but rather concerned lexical semantics: bvFTD patients had difficulties with abstract nouns. Abstract nouns can be less easily experienced perceptually than (objects denoted by) concrete nouns. Moreover, they can be encountered in more variable contexts that concrete nouns. Thus, the common denominator of the abstract noun processing deficit in bvFTD (Cousins et al., 2016, 2017) and the failure to generalize the altered quantifiers semantics (present study) seems to be the dealing with contents which have an abstract representation that cannot be experienced directly and perceptually. This account is commensurable with the model of quantifier acquisition (Sullivan and Barner, 2011) who assume that the acquisition of quantity expressions in children requires learning mechanisms very similar to those needed for learning (concrete) content words. As a limitation, it should be noted that the performance of the patients in the present study was not correlated with neuroimaging data of their atrophy, thus not permitting definite conclusions about the exact brain locus of the performance deficit. In future studies, the individual learning patterns, i.e., the presence of reinforcement learning effects (or their absence) and the lack of a presence of systematic generalization should be plotted directly against the patterns of brain atrophy in structural and also functional neuroimaging protocols.

In contrast, direct reinforcement learning was still possible. Strenziok et al. (2011) reported potential deficits of bvFTD patients also in this field. In the present study, the average MMSE level of the patient group was characterized as “non-demented,” with only four patients falling below the cut-off. Even though there was no direct linear relationship between the MMSE score and the direct learning performance, the patients with MMSE scores below the cut-off fell into the worst-performing quadrant. Given these findings and the fact that the bvFTD patients were still able to perform the truth value judgment task with quantified statements and to show direct reinforcement learning, one may tentatively speculate the following: In the progress of bvFTD, generalization from one concrete instance to a semantically related, but more abstract instance is an ability the patients lose comparatively early – this might be associated with the difficulties of social decorum. Reinforcement learning *per se* might be affected in the next stage as cognitive decline progresses. The ability to process quantities and quantifier statements *per se* may still be preserved in concrete laboratory contexts like the present study when quantifiers and quantities are presented on the screen, but quantities may at the same time lose their reinforcement value in real life (Clark et al., 2018; Wong et al., 2018). This may be associated with the deficit in adapting person-directed behavior to the requirements of the social context, which is also defined by abstract rules (e.g., Zakzanis, 1998; Ibañez and Manes, 2012; Carr et al., 2015) and in which intact meta-cognitive abilities would be required (Eslinger et al., 2005). As stated above, this is, at present, only a speculation which links the observed data to reports in the literature. It would be interesting to pursue the observed effects longitudinally and relate them directly to the behavior of bvFTD patients in different social situations. Finally, Heim et al. (2020) demonstrated that the flexibility of quantifier processing can also be tested with a modification of the original paradigm in which no reinforcement learning takes place. Instead, in the training block (Block 2), the participants only see stimulus pictures showing a limited range of proportions of the named color (e.g., only 20/30/40/50, and not 60/70/80). In line with Helson (1948) adaptation level theory, even this slight modification of the base rate of proportions is sufficient to introduce a change in meaning for the one quantifier present in that block and also a generalization to the untrained quantifier. In future, the application of this modified paradigm to patients suffering from bvFTD could shed further light on the question whether their preserved learning abilities are dependent on reinforcement or not.

The present findings could help caregivers understand that, at least in the earlier phases of disease, patients with bvFTD may be able to learn (simple) tasks when there are well-defined rules, though they also demonstrate that generalization to other situations, even when related, may not take place. In particular, when the acquisition and maintenance of socially adequate behavior is concerned, therapists, caregivers, family and friends might want to reinforce every single concrete behavior consistently. Moreover, they should not expect transfer learning from one situational type to another. Telling the patient in every individual circumstance the type and extent of desired behavior might appear the most promising way to assist the patients in order to provide successful participation and quality of life as long as possible.

## Limitations

One limitation of the present study is the comparably small sample size. Moreover, the age range appears large, potentially adding to inter-subject variability. Finally, the largest proportion of our participants was male. However, we tried to counter these limitations in the following way. First, we sought for robustness and thus representativeness in the performance data of the patients. The comparison of the two experiments yielded the (perhaps for studies with healthy participants not surprising) result that direct reinforcement learning was consistent over patients. In other words, a patient with a large performance shift in one experiment exhibited comparable performance in the other experiment, and vice versa. This shows that the group effects of learning in the two experiments are no random findings, but systematic. In contrast, the lack of a generalization effect to the untrained quantifier, which is presumably due to the brain atrophy in the patients, shows a random distribution in both experiments. This random effect, in turn, is thus systematic. As for age, the demographic data reveal that only one patient (aged 82 years) renders the age range seemingly large. Interestingly, this patient has a MMSE score of 27 and is thus in the upper range of the distribution, clearly not introducing variability by means of low MMSE scores. Likewise, the patients scores in the other neuropsychological tests lie somewhere in the middle of the distribution, thus not adding any extreme values. Finally, with respect to the gender distribution, one might want to keep in mind that the preponderance of FTD patients are male (roughly 5:1; Ratnavalli et al., 2002) so the risk of introducing a bias here is comparably low. Still, for replication studies, larger samples and more homogeneity in the demographic and performance scores would be desirable.

## Data Availability Statement

The datasets generated for this study are available on request to the corresponding author.

## Ethics Statement

The studies involving human participants were reviewed and approved by Institutional Review Board convened at the University of Pennsylvania. The patients/participants provided their written informed consent to participate in this study.

## Author Contributions

SH: study concept, development of study design and stimuli, data analysis, discussion, manuscript. CM: contribution to study design and development of stimuli, recruitment/data acquisition, discussion, correction of manuscript. CO: recruitment/data acquisition, discussion, correction of manuscript. MG: study concept, development of study design and stimuli, discussion, correction of manuscript.

## Conflict of Interest

The authors declare that the research was conducted in the absence of any commercial or financial relationships that could be construed as a potential conflict of interest.
